# Supplementary figures and images for: A high infectious simian adenovirus type 23 vector based vaccine efficiently protects common marmosets against Zika virus infection
Source: PLoS Negl Trop Dis. 2020 Feb 12;14(2):e0008027. doi: 10.1371/journal.pntd.0008027 (PMC7015313; doi:10.1371/journal.pntd.0008027)

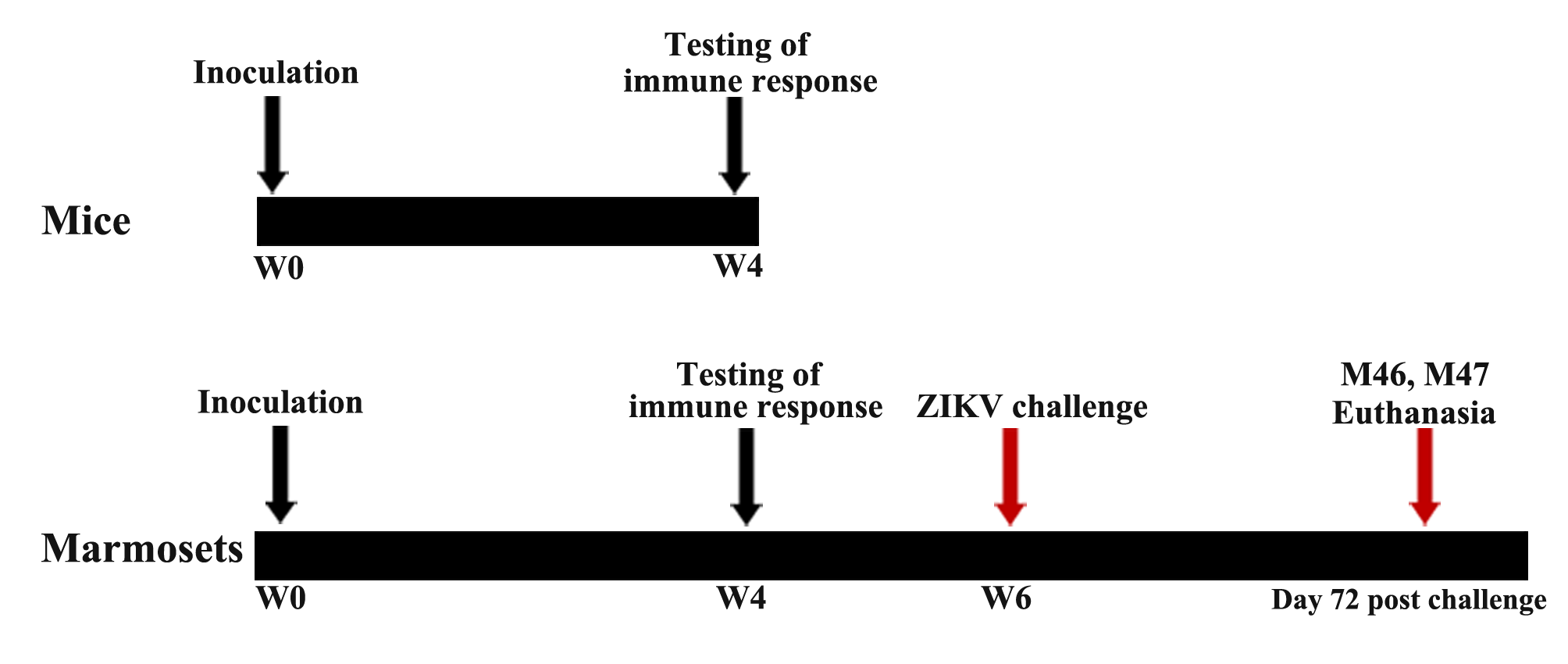

Supplement: S1 Fig — Mice and common marmosets were immunized with Sad23L-prM-E at week 0; evaluation for immunogenicity at 4 weeks post vaccination. Marmosets were challenged with ZIKV at 6 weeks post vaccination; Sham M46 and vaccinated M47 were euthanized at 72nd day post challenge; Sham M48, vaccinated M34 and M37 were persistently monitored. (TIF) [file pntd.0008027.s001.tif]

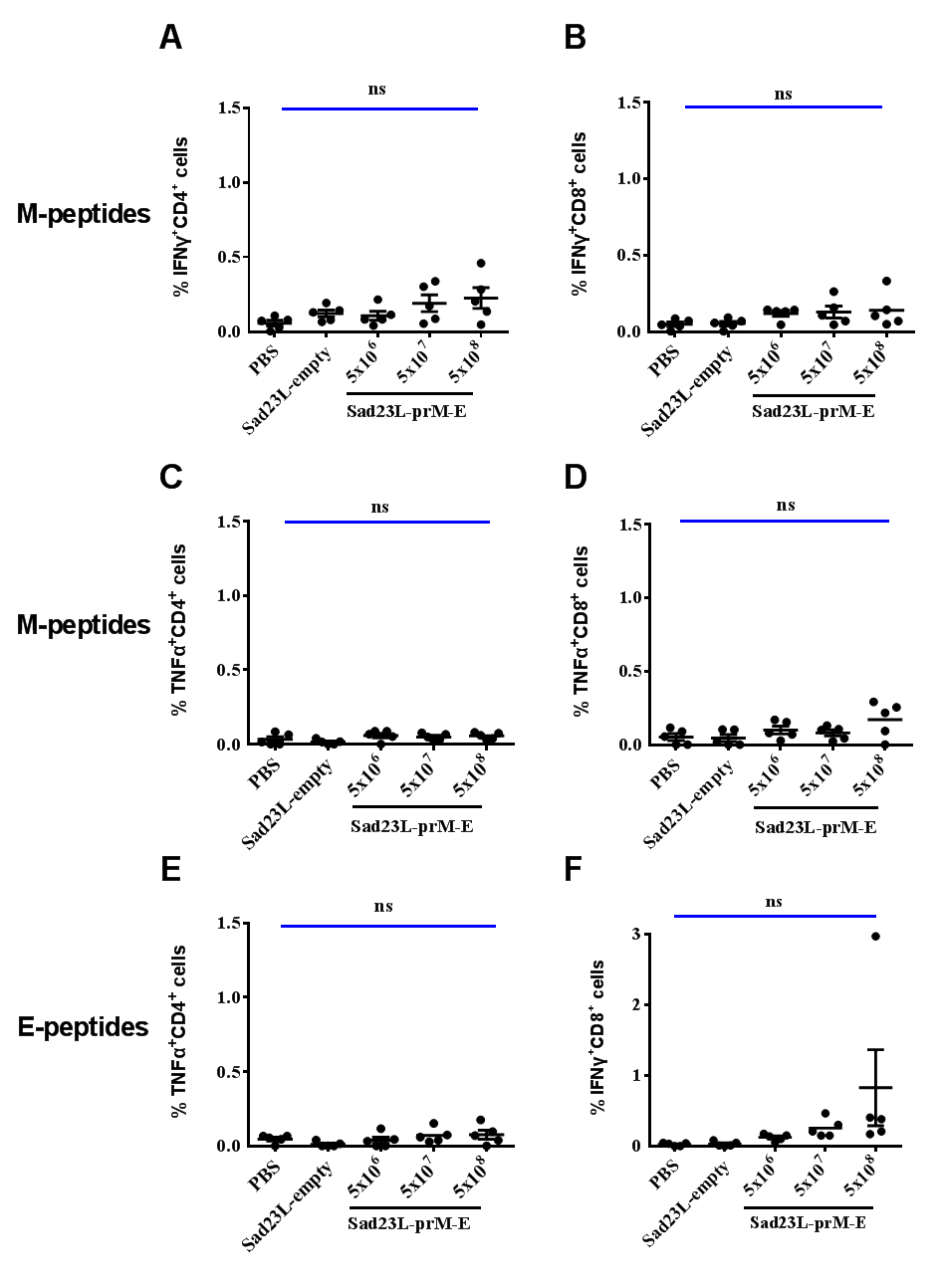

Supplement: S2 Fig — (A-D) The rate of intracellular cytokine+ CD4+ or CD8+ cells of splenocytes to M peptides. (E and F) The rate of intracellular cytokine+ CD4+ or CD8+ cells of splenocytes to E peptides. Data is shown as means ± SEM (standard errors of means). P values are analyzed by one-way ANOVA. Statistically significant differences are showed with asterisks (*, P<0.05; **, P< 0.01 and ***, P< 0.001). ns, P>0.05 and no significant difference. (TIF) [file pntd.0008027.s002.tif]

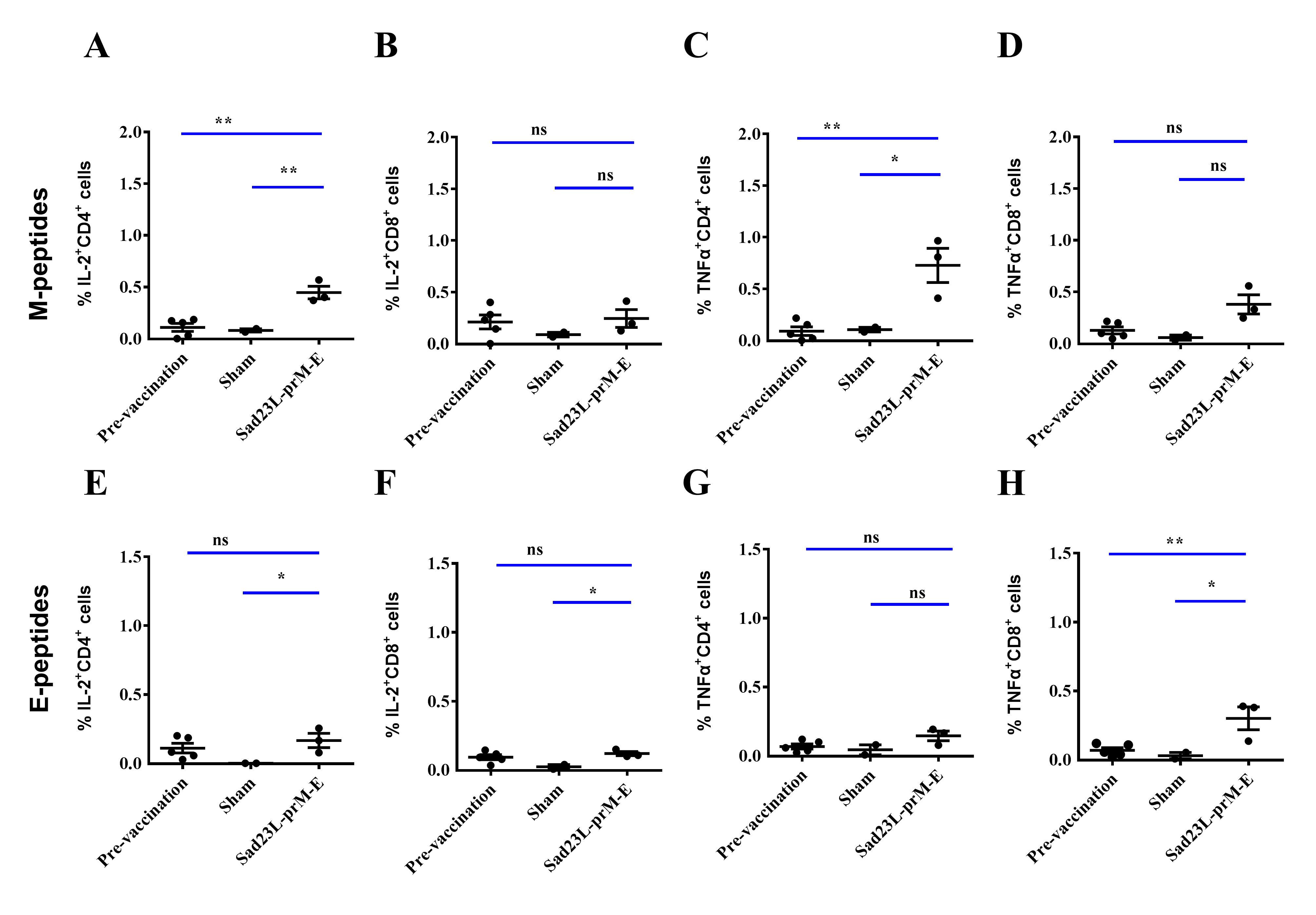

Supplement: S3 Fig — (A-D) The rate of intracellular cytokine+ CD3+CD4+ or CD3+CD8+ cells of PBMCs to M peptides. (E-H) The rate of intracellular cytokine+ CD3+CD4+ or CD3+ CD8+ cells of PBMCs to E peptides. Data is shown as means ± SEM (standard errors of means). P values are analyzed with one-tailed t test. Statistically significant differences are showed with asterisks (*, P<0.05; **, P< 0.01 and ***, P< 0.001). ns, P>0.05 and no significant difference. (TIF) [file pntd.0008027.s003.tif]

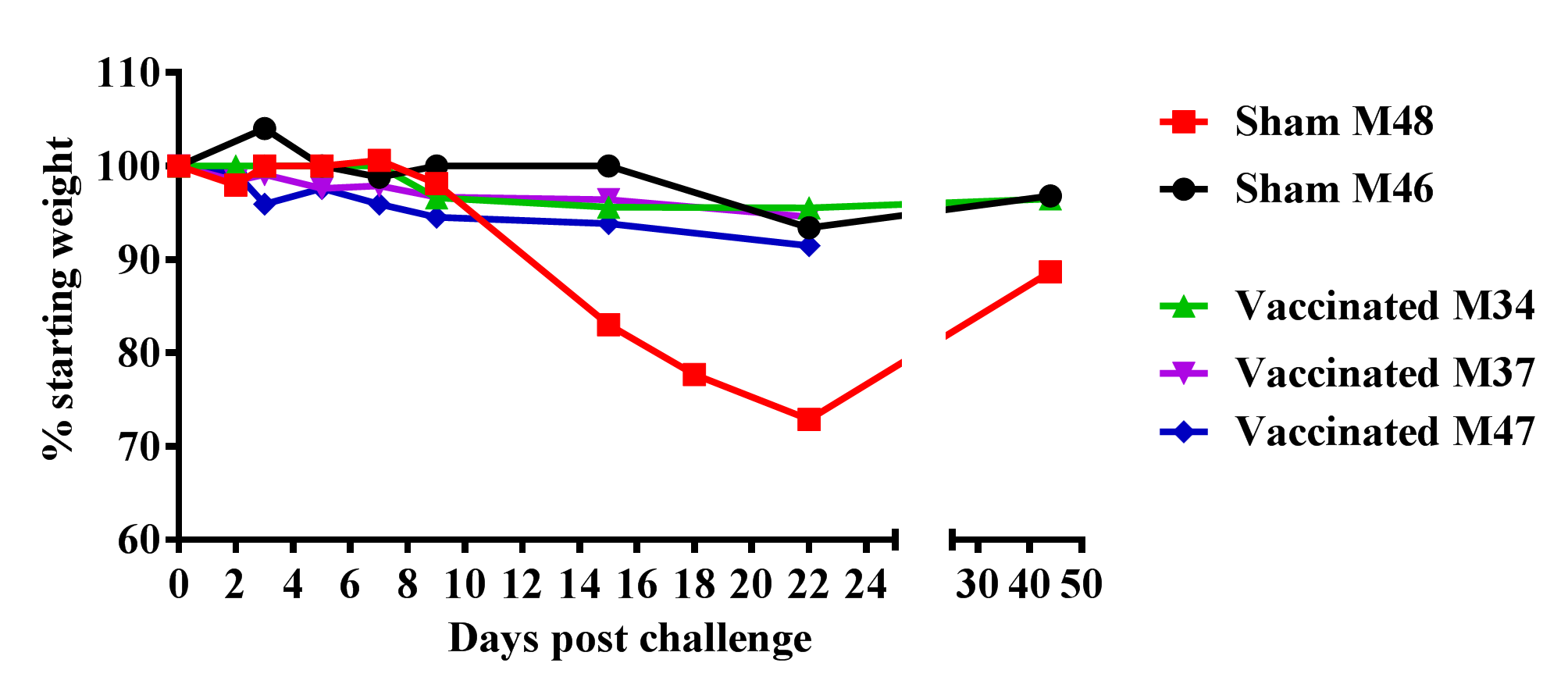

Supplement: S4 Fig — Weight loss was only observed in non-vaccinated M48 and began at 9th day post challenge. The lowest weight was 73% of initial weight at 22nd day, and then went back to 89% of initial weight at 44th day post ZIKV challenge. (TIF) [file pntd.0008027.s004.tif]

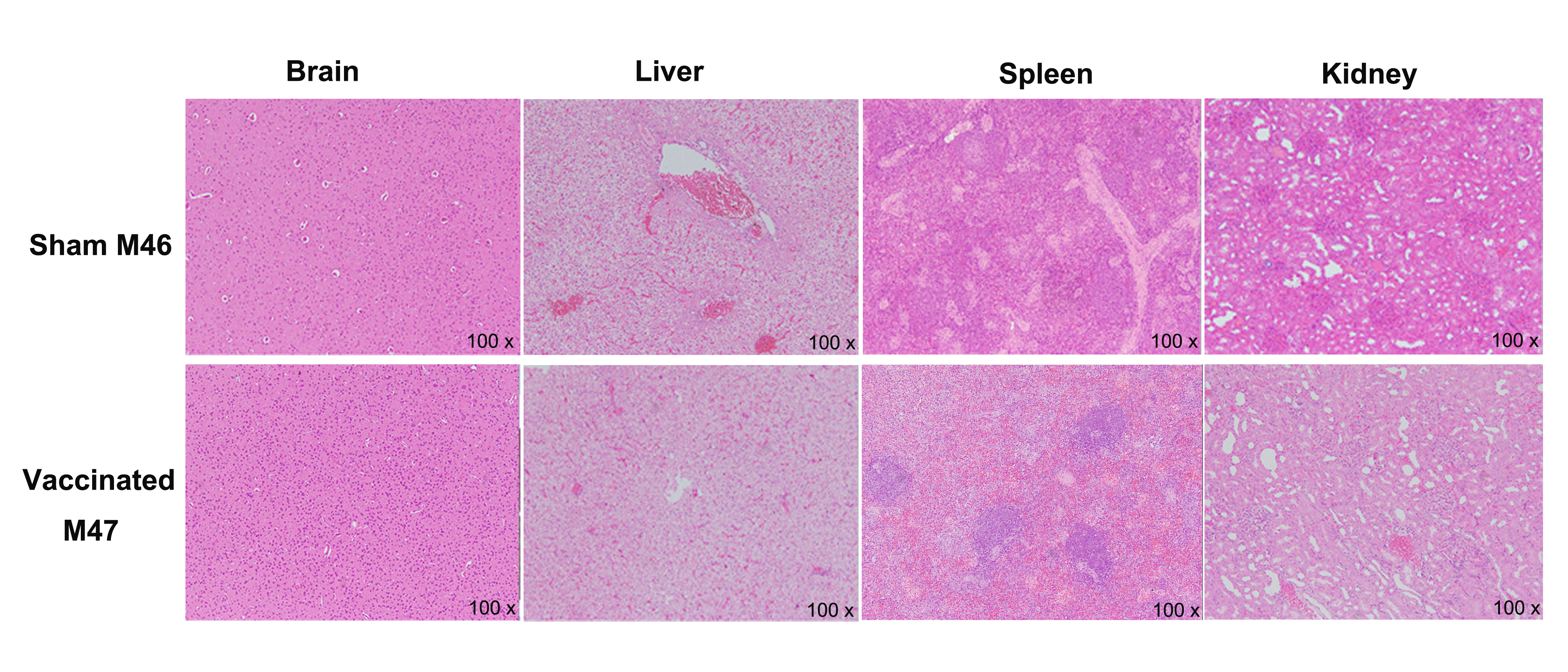

Supplement: S5 Fig — Marmoset tissues were isolated and immediately fixed in 10% buffered formalin solution. The tissues were stained with hematoxylin and eosin (H&E), and examined microscopically for histopathological changes at a magnification of 100 ×. (TIF) [file pntd.0008027.s005.tif]

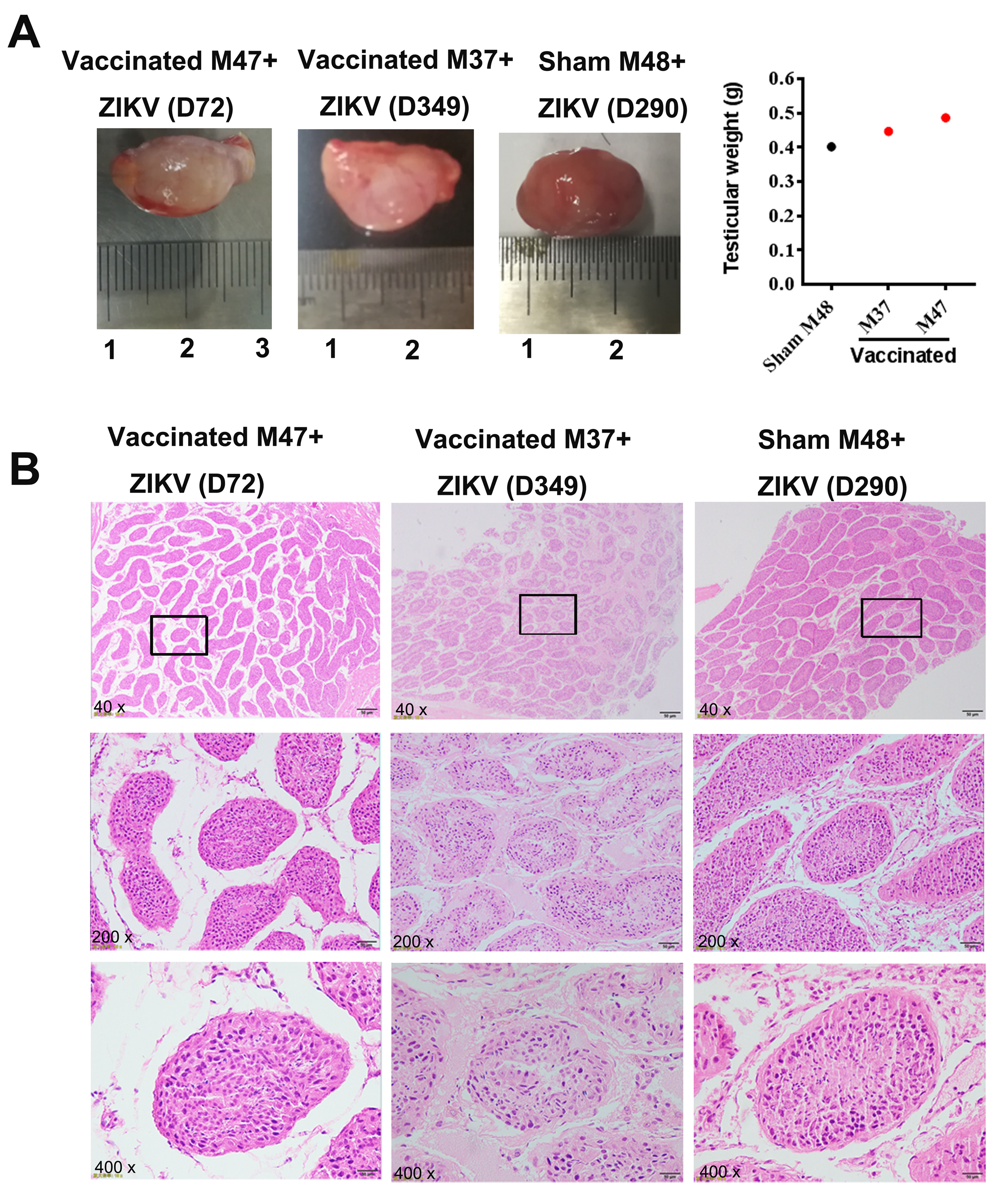

Supplement: S6 Fig — Vaccinated M47 was euthanized at 72nd day post-challenge. Sham M48 and vaccinated M37 died at 290th and 349th day post-challenge, respectively. Marmoset testis were isolated and stained with hematoxylin and eosin (H&E) and examined microscopically for histopathological changes. (A) Testis from marmosets shown in size and weight. (B) Images of testes histopathology. (TIF) [file pntd.0008027.s006.tif]
