# Supplementary material for: A high infectious simian adenovirus type 23 vector based vaccine efficiently protects common marmosets against Zika virus infection
Source: PLoS Negl Trop Dis. 2020 Feb 12;14(2):e0008027. doi: 10.1371/journal.pntd.0008027 (PMC7015313; doi:10.1371/journal.pntd.0008027)
Supplement: S1 Table — (DOCX) [file pntd.0008027.s007.docx]

**S1 Table. Common marmoset immunization regimen.**

| **Group** | **Marmoset**  **(gender)** | **Age**  **(year)** | **Weight**  **(g)** | **Pre-exposure**  **(NAb to Ad5)** | **Pre-exposure**  **(NAb to Sad23L)** | **Immunization (i.m.)** | **Challenge (ZIKV) (i.m.)** |
| --- | --- | --- | --- | --- | --- | --- | --- |
| **Sham**  (PBS) | M46(F) | 4 | 270.00 | <1:10 | <1:10 | 500µl PBS | 1×10^5^ PFU (500µl) |
|  | M48(M) | 4 | 318.03 | <1:10 | <1:10 | 500µl PBS | 1×10^5^ PFU (500µl) |
| **Vaccination**  (Sad23L-prM-E) | M47(M) | 4 | 281.94 | <1:10 | <1:10 | 3×10^8^ PFU | 1×10^5^ PFU (500µl) |
|  | M37(M) | 4 | 329.12 | <1:10 | <1:10 | 3×10^8^ PFU | 1×10^5^ PFU (500µl) |
|  | M34(M) | 5 | 377.66 | 1:40 | <1:10 | 3×10^8^ PFU | 1×10^5^ PFU (500µl) |
