# Supplementary material for: A high infectious simian adenovirus type 23 vector based vaccine efficiently protects common marmosets against Zika virus infection
Source: PLoS Negl Trop Dis. 2020 Feb 12;14(2):e0008027. doi: 10.1371/journal.pntd.0008027 (PMC7015313; doi:10.1371/journal.pntd.0008027)
Supplement: S2 Table — (DOCX) [file pntd.0008027.s008.docx]

**S2 Table. Primers of RT-qPCR or RT nested-PCR.**

| Primers | Sequence (5’-3’) |
| --- | --- |
| ZIKV-NS5-F | GGCRTTRGCCATCAGTCG |
| ZIKV-NS5-R | ATGGAGCATCCGKGAGACT |
| Probe | FAM-TGGCAGCTYCTTTATTTCCACARAAG-BHQ1 |
| Outer Capsid-F1 | ATGAAAAACCCAAAAAAGAAATCCGGA |
| Outer Capsid-R1 | TCATCCAGCATAGGGCATTCATAGCT |
| Inner Capsid-F2 | TTCCGGATTGTCAATATGCT |
| Inner Capsid-R2 | ATGGTGGCATCACACATGTGTC |
| Outer hexon-F1 | ATCGGTCTTATGTACTAC |
| Outer hexon-R1 | GTCCATGGGGTCCAGCGACC |
| Inner hexon-F2 | TCCCAGCTGAATGCTGTG |
| Inner hexon-R2 | TCCAAGGGGAAGCAAT |
| Outer E-F1 | AAGCCTAGGACTTGATTGTG |
| Outer E-R1 | ACGGGGTTAGCGGTTATCAG |
| Inner E-F2 | ACTCCACACTGGAACAACAAAGAAGC |
| Inner E-R2 | GTGAACGCTGCGGTACACAAGGAGTA |
