# Supplementary material for: A high infectious simian adenovirus type 23 vector based vaccine efficiently protects common marmosets against Zika virus infection
Source: PLoS Negl Trop Dis. 2020 Feb 12;14(2):e0008027. doi: 10.1371/journal.pntd.0008027 (PMC7015313; doi:10.1371/journal.pntd.0008027)
Supplement: S3 Table — (DOCX) [file pntd.0008027.s009.docx]

**S3 Table**. **Key reagents and resources.**

| REAGENT or RESOURCE | SOURCE | IDENTIFIER |
| --- | --- | --- |
| Antibodies | | |
| Mouse monoclonal antibody anti-ZIKV E | BioFront Technologies | Cat#BF-1253-25 |
| Anti-mouse IgG, HRP-linked Antibody | Cell Signaling Technology | Cat#7076 |
| Anti-mouse IgG-Alexa Fluor 594 antibody | Thermo Fisher Scientific | Cat#R37115 |
| Anti-Monkey IgG (HRP) | Abcam | Cat#ab112767 |
| PE-Cy7 Anti-Mouse CD3 | BD bioscience | Cat#561100 |
| PE Rat Anti-Mouse CD4 | BD bioscience | Cat#553049 |
| FITC Rat Anti-Mouse IFN-γ | BD bioscience | Cat#562019 |
| V450 Rat Anti-Mouse TNF-α | BD bioscience | Cat#560655 |
| APC Rat Anti-Mouse IL-2 | BD bioscience | Cat#554429 |
| PE-Cy5 Rat Anti-Mouse CD8 | BD bioscience | Cat#553034 |
| PE-Cy7 Mouse Anti-Human CD3 | BD bioscience | Cat#563423 |
| PE Mouse Anti-Human CD4 | BD bioscience | Cat#556616 |
| PE-Cy5 Mouse Anti-Human CD8 | BD bioscience | Cat#555368 |
| FITC Mouse Anti-Human IFN-γ | BD bioscience | Cat#561053 |
| BV421 Mouse Anti-Human IL-2 | BD bioscience | Cat#566273 |
| APC Mouse Anti-Human TNF | BD bioscience | Cat#551384 |
| Bacterial and Virus Strains | | |
| Simian adenovirus type 23 | ATCC | Cat#VR-592 |
| ZIKV strain Z16006 | Originated from the Center for Disease Control and Prevention of Guangdong Province, China | GenBank no. KU955589.1 |
| Chemicals, Peptides, and Recombinant Proteins | | |
| Protein Transport Inhibitor | BD bioscience | Cat#554724 |
| TMB Substrate Solution | Thermo Fisher Scientific | Cat#N301 |
| ZIKV M protein | Sino Biological | Cat# 40543-V02H2 |
| ZIKV E protein | Sino Biological | Cat# 40543-V08B5-100 |
| Critical Commercial Assays | | |
| Super Signal West Pico Plus chemiluminescent substrate | Thermo Fisher Scientific | Cat#34580 |
| eBioscience Intracellular Fix & Perm Set Kit | Thermo Fisher Scientific | Cat#1987589 |
| Mouse IFN-gamma ELISpotPLUS kits | MabTech | Cat# 3321-4APT-2 |
| Human IFN-gamma ELISpotPLUS kits | MabTech | Cat# 3420-4AST-2 |
| High Pure Viral Nucleic Acid Kit | Roche Diagnostic GmbH | Cat# 11858874001 |
| Transcriptor cDNA Synth Kit | Roche Diagnostic GmbH | Cat#4897030001 |
| FastStart Universal Probe Mast | Roche Diagnostic GmbH | Cat#4914058001 |
| Qiagen RNeasy Mini Kit | Qiagen | Cat#74104 |
| Experimental Models: Cell Lines | | |
| Vero cells | ATCC | Cat#CCL-8 |
| HEK 293 cells | ATCC | Cat#CRL-1573 |
| Huh7.5.1 | Generous gift from Professor Yuanping Zhou, Nanfang Hospital, Southern Medical University | N/A |
| Common marmosets’ peripheral blood mononuclear cells (PBMCs) | This paper | N/A |
| Experimental Models: Organisms/Strains | | |
| C57BL/6 mice | Animal Experimental Centre of Southern Medical University, Guangdong, China. | N/A |
| Common marmosets | Tianjin Medical University | NA |
| Software and Algorithms | | |
| Prism 7 software | GraphPad | N/A |
| CFlow Plus Flow Cytometer | BD bioscience | N/A |
