# Supplementary material for: A high infectious simian adenovirus type 23 vector based vaccine efficiently protects common marmosets against Zika virus infection
Source: PLoS Negl Trop Dis. 2020 Feb 12;14(2):e0008027. doi: 10.1371/journal.pntd.0008027 (PMC7015313; doi:10.1371/journal.pntd.0008027)
Supplement: S4 Table — (DOCX) [file pntd.0008027.s010.docx]

**S4 Table. Detection of ZIKV RNA loads in all types of tissues of marmosets post challenge**

| **Tissues**  **(g)**  **RT-qPCR** | Brain  (0.05) | Lymph nodes  (0.03) | Spleen  (0.04) | Liver  (0.05) | Kidney  (0.03) | Testis  (0.05) | Ovary  (0.03) |
| --- | --- | --- | --- | --- | --- | --- | --- |
| **Sham** **M46 (female)** | Neg | Neg | Neg | Neg | Neg |  | Neg |
| **Vaccinated M47 (male)** | Neg | Neg | Neg | Neg | Neg | Neg |  |

Neg, negative for RT-PCR.
