# Supplementary material for: A high infectious simian adenovirus type 23 vector based vaccine efficiently protects common marmosets against Zika virus infection
Source: PLoS Negl Trop Dis. 2020 Feb 12;14(2):e0008027. doi: 10.1371/journal.pntd.0008027 (PMC7015313; doi:10.1371/journal.pntd.0008027)
Supplement: Attachment — Submitted filename: Response to Reviewers.docx [file pntd.0008027.s011.docx]

**Reviewer #1:** The manuscript is easy to follow and the data are explained in the figures and tables within the main text and supplement. I have a few minor comments:
1. Comment on choice of animal sex for the study: all mice were female but marmosets were male (with the exception of one control).
**Answer:** The inbred mice were chosen for female because they were much gentler and easier for handling. The resource of marmosets was very limited in the Experimental Animal Center of Tianjin Medical University where the marmosets were provided for us by its consideration about animal’s sex, and we had no much more choice except for animal’s age and weight. The numbers for use of marmosets were strictly restricted as well by the university ethics committee and five marmosets were approved for this study. In order to minimize the impact of gender in the experiment, this female marmoset was used in the control group.

2. Please provide additional information regarding the JE signal peptide used. Was the sequence from an attenuated JE virus?

**Answer:** Thank you for your suggestion. The JE signal peptide sequence "MGKRSAGSIMWLASLAVVIACAGA” was added in the Materials and methods of revised manuscript (Page 7 and Lines 132-134). The sequence was derived from an attenuated JE virus, for which the JE signal DNA sequence 5’-ATGGGCAAGAGGTCCGCTGGATCCATTATGTGGTTAGCATCCTTAGCTGTCGTGATTGCATGTGCAGGCGCA-3’ was synthesized by Beijing Genomics Institute, Beijing, China.

3. Why was strain Z16006 selected for the study? What is its source and history?

**Answer:** Z16006 strain (GenBank no. KU955589.1) was an Asian lineage and predominant in the Southern America and the Southeast Asia. This strain was isolated by the Center for Disease Control and Prevention of Guangdong Province, China on 16 February 2016 from a patient who travelled to Fiji and Samoa where it happened to be the epidemic areas of ZIKV. The more detailed information has been added in the revised manuscript (Page 6 and Lines 116-119).

4. Why was the dose of 3x10^8 pfu selected for vaccination?

**Answer:** According to the determination of immunogenicity of vaccine in mice, the principle of “necessary and sufficient” and the weight ratio of marmoset to mouse, a dose of 3x10^8^ PFU Sad23L-prM-E vaccines was used for vaccinating marmosets. Based on 5x10^7^pfu as the optimal dose in mice, considering the weight of marmoset (300g) is six times to the mouse (50g), the vaccination dose of marmoset was calculated as 3x10^8^ pfu. The criterions were added in the Materials and methods of revised manuscript (Page 9 and Lines 164-166).

5. The adult marmoset model is for subclinical disease; therefore, it limits the ability to test whether or not the vaccine reduces disease.

**Answer:** Thank you for your question and comment. According to our results from vaccinated or sham marmosets challenged with ZIKV, viral persistence was observed in blood and body fluids in sham controls, while no viral nucleic acid was detected in tissues and no significant gross pathological lesions were observed in any of the post-mortem tissues excepting for light pathological changes in the testis of a male control marmoset examined at 290^th^ day post infection. It was similar with the reference “Chiu CY et al. Experimental Zika Virus Inoculation in a New World Monkey Model Reproduces Key Features of the Human Infection. Sci Rep. 2017. 7(1):17126”, in which four male marmosets were infected with ZIKV and appeared RNA in blood and body fluids, with no significant gross pathological lesions were observed, and only one was detected virus (3,680 copies/mg) in lymph node tissue. The adult marmoset model was for subclinical disease with viremia, which might limit the ability to test the vaccine efficacy against disease in a short period, but a long-time observation found the light pathological changes in the testis from a sham marmoset (M48) infected with ZIKV (Page 21 and lines 409-414; S6 Fig), and it was suitable to evaluate the vaccine induced immune response.

6. Comment on the weight loss of only one of two sham marmosets. Is the weight loss considered significant in these animals? Were there any other major differences in the response to the challenge between the two sham marmosets?

**Answer:** The weight loss was found for a male marmoset M48, but not for another female marmoset M46, therefore it was hardly considered significant for the other animals. However, M48 with temporary weight loss was observed for light pathological changes in weight and tissues of testis at 290^th^ day post infection, while other three vaccinated male marmosets including examining of testes tissues of M47 and M37 were no significant changes after ZIKV challenge (see newly added S6 Figure and the corresponding description in Page 21 and lines 409-414).

7. The methods state that viral genome in marmoset tissues was assessed in brain, testis, lymph nodes, spleen, liver, and ovary, but data from all of these tissues are not shown. Please revise accordingly.

**Answer:** Thank you very much for your suggestion. ZIKV RNA was extracted from the tissues of sham M46 and vaccinated M47, and quantified by RT-qPCR, but all tissues were detected negative. The additional descriptions were presented in new S4 Table of supplementary information, which was addressed in Page 20 and line 407.

8. No H&E images are included in the manuscript. Was any histopathology expected in these animals?

**Answer:** Thank you for your comment. In previous manuscript, the images were not included, as no significant gross pathological lesions were observed in any of the post-mortem tissues except for light changes in testis from a male sham animal. In the new version of revised manuscript, the pathological and histopathological observations for all types of tissues of animals including testis from two vaccinated and one sham male animals challenged with ZIKV were newly presented in S5 Figure and S6 Figure of supplementary information, respectively. The additional descriptions were added accordingly in the Results section of revised manuscript (Page 20-21 and lines 408-414)

9. Please edit the manuscript for typographical and minor language errors. Examples:
“images” not “photos” 
Line 453: “Decreased” not “disappeared” 
“are shown” not “are showed” 
The “10” base is missing after “log” in the y-axes

**Answer:** Thank you very much for your corrections. All corrections have been made in the revised manuscript. “Disappeared” was corrected in Page 25 and Line 507. All “are showed” were corrected as “are shown”. All figures of Log in the Y-axes were corrected with Log10.

10. The statistical methods used for each assay should be clarified. Specifically, two-tailed tests are used throughout the study, but are these appropriate for all of the comparisons? One-tailed tests carry more power. Also, please clarify which specific values are included in the comparisons.

**Answer:** We thank the reviewer for this suggestion. Detailed statistical methods were included in the figure legends according to different situations. At the same degree of freedom, the *p* value of two-tailed tests is two folds than that of one-tailed. Indeed, one-tailed tests carry more power, while on the other hand, the type one error (the false positive probability) would increase using one-tailed tests. In order to be sure about the reliability of the results for the significant difference between the vaccinated group and the control group, we chose two-tailed tests in a conservative way.

The Figure 4 legend states that the values (points) in Figure 4 I-L represent the peak titers in the preceding panels. What are the p-values in Figure 4 E-K comparing?

**Answer:** Figure 4 I-L were comparisons of the peak viral loads between sham and vaccination by two-tailed *t* tests. P values in Figure 4 E-H were comparisons of all values between sham and vaccination groups by two-tailed Mann-Whitney U tests, which were added in the replotted Figure 4 E-K (Page 41).

Figure 5- what is being compared to arrive at the p-values in the graphs?

**Answer:** Neutralizing antibody titers (NAb) and T cell response (E-peptides) between sham and vaccination groups were compared by two-tailed Mann-Whitney U tests, and the *P* values were presented in Figure 5B and 5D, respectively. This description has been added in the revised Figure 5 legend accordingly (Page 43-44).

Fig 4 – It seems that 2log10 is the limit of detection, so what does the dashed line at 3log10 represent?
**Answer:** The dashed line at 3 log10 indicated a relatively low level of viral load, which was added in the Fig. 4 legend (Page 42 and lines 790-791).

**Summary and General Comments**

**Reviewer #1:** In the manuscript the authors use a simian adenovirus vector vaccine, Sad23-prME, to immunize mice and marmosets against the prME of Zika virus in order to overcome the lower yield issues of another adenovirus vectored ZIKV vaccine using RhAd52. Vaccination of marmosets led to immunity against ZIKV and reduced viral loads (viral RNA) detected in saliva and blood samples following challenge with an Asian ZIKV strain. 

Weaknesses: 
Vaccination did not provide sterilizing immunity, and the narrow spectrum of disease in the marmoset model rather limits assessment of the vaccine’s ability to protect from symptomatic disease that could occur following ZIKV infection. Further, only male marmosets were vaccinated, and it remains to be seen whether female marmosets generate similar response that can protect in a pregnancy model. 

Strengths: 
The authors generated a new vaccine for ZIKV. Immunity in mice was shown prior to moving to NHP experiments. The authors performed a comprehensive set of experiments, many of them with serial sampling, and a wide-range of assays to show that Sad23-prME vaccine elicits antibody and cellular immunity against ZIKV.

**Answer:** We are thankful for the reviewer’s pertinent comments. Whether female marmosets generate similar response that can protect in a pregnancy model will be included in the next step.

**Reviewer #2:** Luo et al present a study to highlight the efficacy of a recombinant simian adenovirus serotype 23 vector encoding PrM and E against Zika virus and the utility of marmosets as a challenge model to assess Zika virus vaccine efficacy. Whilst the study presents useful results to advance the knowledge base for Zika virus vaccine development, there are several caveats about the study that need to be addressed:
1) The marmoset vaccine efficacy study does not appear to be sufficiently powered to generate statistically significant results. I am not sure how the authors show p values (less than 0.05) when the sham group only has 2 marmosets for comparison purposes against the vaccinated group.

**Answer:** Thank you very much for your critical comment. For statistical analysis, the larger the number of samples is, and then the more reliable the results are. With the present 2 marmosets for sham group, appropriate statistical analysis was also practicable to be performed. Additionally, to make up for this, pre-vaccination results were added as the reviewer suggested in the following, which was helpful to present a clear difference for comparison.

2) There is no pre-bleed data presented for the marmoset study to establish a reasonable baseline for T cell and antibody responses for each marmoset. Given that these are I presume, outbred marmosets unlike inbred mice, this data is very relevant for comparison (pre-bleed vs post-vaccination bleeds) unless the authors can justify otherwise.

**Answer:** Thank you very much for your suggestion. Indeed, we carefully detected baseline data in the pre-bleed samples from all five marmosets before the immunization experiments, including neutralizing or reactive antibody levels, specific CD4+/CD8+ T cell responses with various cytokines by ELISpot and flow cytometry, respectively. According to your suggestion, these baseline data have been added as a group in the replotted Fig 3 for comparisons of pre-bleeds vs post-vaccination bleeds from vaccine or control groups. The corresponding descriptions have been added in the Results section (Page 16-17 and lines 320-328), and the replotted Fig. 3 legend of revised manuscript (Page 39 and Line 752-759). The comparisons with pre-vaccination group were addressed accordingly (Page 17-18 and lines 336, 338, 342-346, 349-352, 356, 359-364).

3) The frequency data for T cell responses is not convincing because proportion analysis is susceptible to skewing effects. The authors should show representative flow cytometry plots for the ICS analysis in figure 2 and show the absolute cell numbers to show the total number of cytokines producing cells for all the ICS data in the manuscript. Statistical significance comparison in figure 2 should compare all groups analyzed and not just the highest dose.

**Answer:** Thank you for your comment and suggestion. The representative flow cytometry plots for all panels were added accordingly in the replotted Fig 2. The descriptions were added in the replotted Figure 2 legend (Page 36 and line 739) and the corresponding descriptions regarding the reactive rates (numbers) for various cytokines positive cells from three doses (5×10^8^, 5×10^7^ and 5×10^6^ PFU) of vaccines immunized mice verse Sad23L-empty and PBS sham mice were added accordingly in the Results section of manuscript (Page 15-16 and lines 298-311). According to your suggestion, *P* values were newly added in the replotted Figure 2 for all groups of comparisons.

4) Why were the marmosets challenged using the intramuscular route? The data regarding which route is most robust in establishing Zika virus infection of marmosets is lacking. Studies using rhesus macaques show infectivity of Zika virus using the subcutaneous route and is the route used to evaluate vaccine efficacy in macaques even in the relevant papers the authors have cited.

**Answer:** Thank you for your comments. As reviewer mentioned, rhesus macaques were experimentally infected by ZIKV mostly via subcutaneous route in previous studies. However, the recent publication by Chiu CY et al used the intramuscular injection of ZIKV successfully to infect four male marmosets (Experimental Zika Virus Inoculation in a New World Monkey Model Reproduces Key Features of the Human Infection. Sci Rep. 2017. 7(1):17126). As we know that intramuscular injection route is more conducive for rapid introduction of virus or vaccine into the circulation blood. If the vaccine raised immune response was powerful enough, the established immunity would clear the challenging virus rapidly. So we decided to challenge the vaccinated and sham marmosets by ZIKV injection with intramuscular route. We agree with reviewer’s comment that the most robust infection route should be defined by comparing with subcutaneous route. However, as the marmoset was classified in second class of protected animals in China and the limited numbers of marmosets for this study were approved by the ethics committee, and in addition the cost of marmoset was very expensive (about 5000 US dollars per animal), we didn’t try other routes for challenging marmosets by ZIKV. The reason of transient and low viremia appeared in vaccinated marmosets might be related to the intramuscular route used, as a large amount of ZIKV rapidly entered into the blood but were rapidly cleared.

5) Why were marmosets vaccinated using 3 x 108 PFU of the vaccine when in mice a dose of 5 x 108 PFU elicited the highest immune responses? Based on the mice data, the authors should have used a higher dose which may have resulted in better protective and immune outcomes especially given that some of the T cell responses (i.e. IL-2 and TNF production) in vaccinated marmosets were comparable to the sham control.

**Answer:** According to the results of immunogenicity in mice with the principle of “necessary and sufficient” and the toxicity of the adenoviral vaccine, we selected 5x10^7^pfu as the optimal dose in mice. Considering the weight of marmoset (300g) is six times to the mouse (50g), the vaccination dose of marmoset was calculated as 3x10^8^ pfu. The criterions were added in the Materials and methods of revised manuscript (Page 9 and Lines 164-166).

6) The methods section needs to be more descriptive. For example, how many cells were stimulated using peptides? How were the cells cultured and serum added in the PRNT assay? What is composition of ‘complete’ DMEM? If the virus was propagated using HEK 293 cells, how many passages can the recombinant virus be cultured for? The clones of the monoclonal antibodies including those used for ICS should be mentioned.

**Answer:** Thank you for your suggestion. We made modification by adding more descriptions highlighted in yellow in the Materials and methods section of revised manuscript accordingly for the numbers of cells, please see Page 10 and lines 193-200; for complete DMEM, please see Page 7 and lines 121-123; for PRNT assay, see Page 9 and lines 181-187; for vaccine strain passage, see Page 7 and lines 138-139; for antibodies used for ICS, see Page 11 and Lines 204-208, and more detailed information were presented in the supplementary S3 Table.

7) There are some typos and the second last sentence of the Abstract (starting at line 40) should be re-written because it is confusing. In line 269, replace the word ‘induction’ with stimulation. The abbreviations for IL-2, TNF and IFN need to be spelled out before they are abbreviated initially.

**Answer:** Thank you very much for your corrections. We carefully revised the text and all pointed typos were corrected, including the conclusion sentence in the Abstract (Page 2 and Lines 40-42). The “induction” was replaced with “stimulation” (Page 15 and Line 292), and the full names of IL-2, TNF-α and IFN-γ were spelled out before the abbreviations (Page 11 and Lines 207-208).
