# Supplementary material for: A high infectious simian adenovirus type 23 vector based vaccine efficiently protects common marmosets against Zika virus infection
Source: PLoS Negl Trop Dis. 2020 Feb 12;14(2):e0008027. doi: 10.1371/journal.pntd.0008027 (PMC7015313; doi:10.1371/journal.pntd.0008027)
Supplement: Attachment — Submitted filename: Response to Reviewers.doc [file pntd.0008027.s012.doc]

**Reviewer #1**: The authors addressed the comments in the initial review. However, they should perform one-tailed tests and provide the actual p-values, even inf they're greater than 0.05. This is the accepted statistical method to use. A vaccine should increase antibody titers and reduce viremia. Assuming that the data distribution could be two-tailed would imply that titers can be less than zero, which they cannot.

**Answer:** We accepted reviewer’s suggestion. All relevant comparisons were re-calculated by one-tailed tests. The newly obtained *P* values were added in the revised manuscript and the re-plotted figures correspondingly by replacing previous two-tailed *P* values. The changes were highlighted in yellow in this new version of manuscript (Lines 335, 337-338, 343-346, 350-352, 355-366, 384, 389-393,430, 436).

**Reviewer #1:** Some figure panels are very small and could be enlarged.

**Answer:** Figure panels Fig2E-N, Fig3, Fig4A-L and Fig5 were enlarged in newly plotted ones.
